# Modeling cancer using patient-derived induced pluripotent stem cells to understand development of childhood malignancies

**DOI:** 10.1038/s41420-017-0009-2

**Published:** 2018-02-01

**Authors:** Ana Marin Navarro, Evelyn Susanto, Anna Falk, Margareta Wilhelm

**Affiliations:** 1 Department of Microbiology, Tumor and Cell biology (MTC), Karolinska Institutet, Stockholm, Sweden; 2 Department of Neuroscience, Karolinska Institutet, Stockholm, Sweden

## Abstract

In vitro modeling of complex diseases is now a possibility with the use of patient-derived induced pluripotent stem (iPS) cells. Their stem cell properties, including self-renewal and their potential to virtually differentiate into any cell type, emphasize their importance as a translational tool for modeling disorders that so far have been limited by the unavailability of primary cell lines, animal models, or inaccessible human materials. Around 100 genes with germline mutations have been described to be responsible for cancer predisposition. Familial cancers are usually diagnosed earlier in life since these patients already carry the first transforming hit. Deriving iPS cells from patients suffering from familial cancers provides a valuable tool for understanding the mechanisms underlying pediatric cancer onset and progression since they require less mutation recurrence than adult cancers to develop. At the same time, some familial mutations are found in sporadic cases and are a valuable prognostic tool. Patient-derived iPS cells from germline malignancies can also create new tools in developing specific drugs with more personalized-therapy strategies.

## Introduction

The discovery in 2007 by Yamanaka and colleagues that a combination of just four transcription factors (TFs), Oct4, Sox2, Klf4, and c-Myc (OSKM), were able to revert adult human somatic cells back to pluripotency have had a huge impact on basic research, regenerative research, and cancer research^1,2^. Together with John Gurdon’s pioneering experiments during the 1960’s^3^, it demonstrated that mature and fully differentiated cells can be reprogrammed into a pluripotent state (similar to embryonic stem (ES) cells) with the potential to differentiate into any cellular lineage. Reprogramming of human somatic cells to induced pluripotent stem (iPS) cells overcomes many of the ethical and technical limitations of ES cells, and iPS cells can easily be generated from a tissue biopsy, blood cells, or tumor sample from virtually any person with or without a diagnosis^4^. iPS cells express similar markers as ES cells, they are capable of self-renewal, and importantly, are able to differentiate in vitro and in vivo into cell types of all three germ layers, thus giving rise to a diverse panel of cells. The unlimited supply of disease-relevant cells have made iPS cells a great tool for studying human diseases, especially for those that previously have been restricted to postmortem samples due to inaccessibility of patient material.

Until now most disease models using iPS cells have focused on diseases caused by mutations in a single gene, with early disease onset, and often with high penetrance, such as Fragile X syndrome^5^ and Familial dysautonomia^6^, both monogenic Mendelian diseases, as well as chromosomal diseases such as Down’s syndrome^7–9^. However, most diseases are not hereditary but sporadic and are genetically complex with mutations at multiple loci and have a late onset with low penetrance, thus considered much more challenging to model.

Nevertheless, complex diseases with sporadic occurrence and late onset have successfully been modeled using disease-specific iPS cells, where vast majority includes neurodegenerative diseases^10–12^. However, these are not the only challenges our increasingly aging society face, but also other common pathologies such as cancer and cardiovascular disorders.

Cancer, is among the leading cause of death after cardiovascular diseases in developed countries, firmly linked not just to genetics but also environmental factors and especially due to the increasing age of the population. Since cancer is a multi-step disease and many stages occur before the actual malignancy is developed and detected, there is an urge to understand the genetic mechanisms that are altered from onset to progression of disease. Mouse models of cancer have brought extremely insightful information in terms of understanding cancer progression mechanisms. However, many therapeutic drugs that have shown excellent efficacy in mouse models have failed in human clinical trials^13^. This shows the need for developing new models based on disease-relevant human cells to identify the right biomarkers useful for treatment. Most human cancer cell models used to date are based on immortalized cancer cell lines and xenografts studies using cells from established tumors. While these techniques are important for understanding mechanisms operating in late-stage tumor development, they will not model tumor initiation and early progression. Therefore, focusing on end point events of the disease might have given us false positives of what changes are actually driving the disease. Moreover, it may also obscure potential early biomarkers that could be translated into the clinic. Using iPS cells for modeling cancer onset and progression could overcome the disadvantages with current techniques and give us new important insight into tumor initiation and development.

## Reprogramming of cancer cell lines

**Fig. 1:**
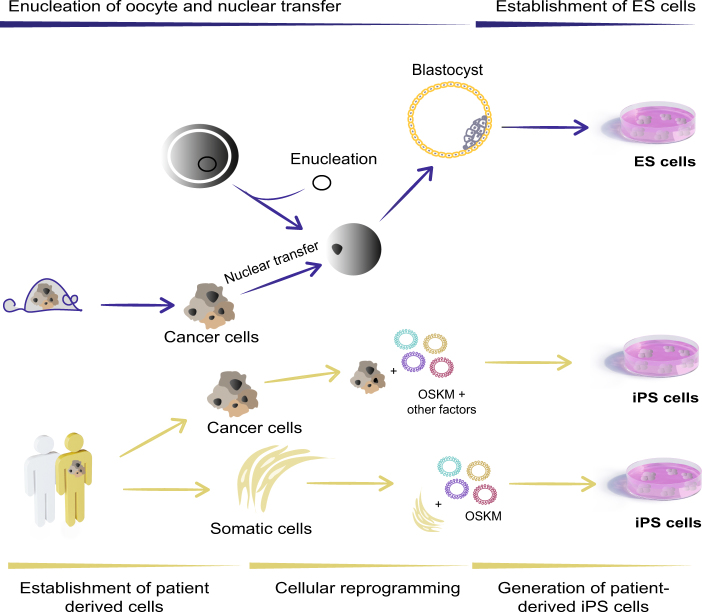
Approaches to generate pluripotent stem cells from cancer cells (Upper part) Somatic cell nuclear transfer (SCNT) leads to reprogramming of the cancer cell nucleus. The nucleus of a cancer cell is microinjected into an enucleated mouse oocyte that further develops into a blastocyst. ES cells are isolated from the inner cell mass of the blastocyst. (Lower part) Due to ethical limitations involving using human pre-implantation embryos, reprogramming using the four Yamanaka factors (OSKM) has been extensively used for human cancer cell lines and somatic cells derived from patients. Retrovirus, Sendai virus, exosomes, and mRNA^85^ among other techniques can be used to deliver the factors and thereafter generate unlimited source of patient-derived iPS cells. ES embryonic stem, iPS induced pluripotent stem, OSKM Oct4, Sox2, Klf4, and c-Myc transcription factors

The emerging need of new and improved cancer models has accelerated the optimization of cancer cell line reprogramming. So far, two different ways for reprogramming somatic cells to pluripotency have been described, by somatic cell nuclear transfer ((SCNT) implantation of a somatic nucleus cell into a enucleated oocyte)^3,14^ or by using ectopic expression of TFs to produce iPS cells (Fig. 1). Whereas just a subset of cancer cells have been successfully reprogrammed by SCNT, the second approach has been more successful since there is no need for the use of oocytes or blastocysts and also due to increased reprogramming efficiency^15^. Whether these differences are due to technical issues or cancer-tissue specificity is still not understood. Despite the challenges, the first report showing successful reprograming of human malignant cells appeared in 2010 when iPS cells were generated using retroviral delivery of OSKM into a chronic myeloid leukemia (CML) cell line, KBM7^16^. Injection of the resulting CML-iPS cells subcutaneously into NOD-SCID mice produced teratomas that contained cells from all three germ layers. The CML-iPS cells were able to differentiate in vitro into cells that express CD43 (pan-T cell marker), CD45 (hematopoietic lineage marker), as well as the stem cell marker CD34, indicating a restoration of differentiation ability into hematopoietic lineages. The parental CML cell lines were dependent on the BCR–ABL pathway; however, the CML-iPS cell lines showed resistance to imatinib, an inhibitor of BCR–ABL signaling, despite expressing the *BCR–ABL* gene. The CML-iPS cells regained sensitivity to imatinib when differentiated in vitro to hematopoietic lineage cells, suggesting that oncogenic dependency of the CML-iPS cells depended on differentiation status of the cells^17^.

**Fig. 2:**
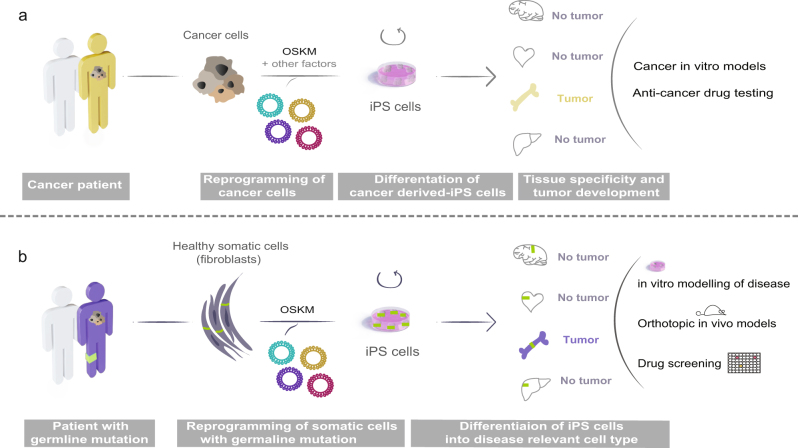
Cellular reprogramming of cancer and somatic cells **a** Cancer cells isolated from patients can be reprogrammed into iPS cells using the Yamanaka factors (OSKM). Thereafter, cancer-derived iPS cells can be differentiated into diverse relevant cell types for studying progression of the disease. The drawing exemplifies how cancer cell-derived iPS cells can be used to study tumor specification, develop in vitro cancer models and testing of novel potential targets for therapy. **b** Cancer patients carrying familial cancer predisposition mutations can be useful for understanding onset and tissue specificity of the disease. Somatic cells can be used for reprogramming into iPS cells that can be differentiated into any relevant cell type. This permits the development of in vitro and in vivo systems for modelling disease and use for potential identification of therapeutic targets. iPS induced pluripotent stem, OSKM Oct4, Sox2, Klf4, and c-Myc transcription factors

Thereafter, other reports verified generation of iPS cell lines from different cancer cells lines or primary tumor cells; melanoma^18^, gastric cancer^19^, glioblastoma^20^, and pancreatic ductal adenocarcinoma (PDAC)^21^, among others. Not all reports have described the use of iPS as a model for cancer but solely the success of using this technique to generate pluripotent cells. However, some studies have gone further and showed the use of cancer cell-derived iPS cells to model the disease (Fig. 2a). Since PDAC lacks an early disease progression model, Kim et al. generated iPS cells from PDAC tumor samples that upon differentiation underwent early stages of pancreatic cancer. When injected into immunodeficient mice, the iPS cells formed intra-epithelial neoplasias (PanINs) which progressed to more invasive stages. Furthermore, they generated and studied pancreatic organoids from the PanIN cells to identify biomarkers and pathways useful for early detection of the disease, since a major problem in diagnosing pancreatic cancer is the lack of symptoms until late-stage disease. The HNF4a network was discovered to be significantly activated in early to intermediate stages of PDAC development, indicating this method may be useful for identifying novel targets for diagnosis and treatment^21^.

However, it has been demonstrated that reprogramming cancer cells can be challenging and some cell lines need other factors such as NANOG, LIN28, BCL2, or KRAS in addition to the classical OSKM factors. The reprogramming problems might originate from the presence of genomic instability, including accumulation of mutations and DNA damage, and epigenetic modifications^22^.

Cancer is not solely driven by genetic defects, extensive research has identified epigenetic changes in cancer cells^23^. Considering that cell transformation, cellular reprograming, and pluripotency are related concepts strongly linked to epigenetic regulation, it has been suggested that reprogramming could epigenetically reset cancer cells. Zhang et al. could show that the sarcoma cell lines SAOS2, HOS, MG63, SW872, and SKNEP could be reprogrammed into iPS cells, which were capable of generating connective tissue and hematopoietic cells^24^. When sarcoma-derived iPS were differentiated into osteogenic and adipogenic lineage and followed by in vivo subcutaneous transplantation into mice, no tumor growth was observed, suggesting reprogramming could overcome the tumorigenic capacity of the parental sarcoma cells^24^. In the same manner, non-small cell lung cancer cell lines were reprogrammed and reversion of their epigenome and transcriptome resulted in reduced tumorigenicity^25^. Therefore, because of the extensive epigenetic remodeling that oncogenes and tumor suppressors undergo via reprogramming, this method has been proposed as a therapeutic strategy. However, it has also been shown that the same approach can lead to the opposite phenotype, resulting in a more invasive and aggressive disease and even resistance to currently available inhibitors, such as seen in PDAC, juvenile myelomonocytic leukemia (JMML), and CML^19,21,26,27^. Even though ectopic expression of pluripotency genes such as reprogramming factors can be achieved using non-integrating techniques^28^, we have to take in consideration that the OSKM factors are also implicated as potent cancer drivers. In addition, it has been suggested that incomplete reprogramming could lead to cancer development. Using a mouse model with inducible OSKM factors, Ohnishi et al. ^29^ showed that long-term (4 weeks) in vivo activation of OSKM factors resulted in reprogramming and teratoma formation in vivo, however, a shorter late activation in vivo for 3–9 days resulted in tumor development in various somatic tissues consisting of undifferentiated dysplastic cells and global changes in DNA methylation patterns. The tumor cells could be reprogrammed into iPS cells by OSKM activation for 2 weeks in vitro. When the iPS cells were injected into blastocysts, they gave rise to non-neoplastic normal kidney cells in the chimeric mice^29^. These findings suggest that epigenetic processes associated with iPS cell reprogramming may also drive cancer development and that this genetic transformation is reversible. Consequently, taking this approach as a potential treatment has to be carefully revised. Due to tumor heterogeneity we might be selecting for clonal subpopulations explaining the discrepancy of reports whether reprogramming cancer cells may alleviate the tumorigenic potential or not.

## Modeling cancer using iPS cells carrying cancer-predisposing germline mutations

Cancer could also be modeled in vitro without the need of reprogramming cancer cells but rather by using non-cancerous cells from patients suffering from familial cancers. Although germline mutations are rare, most of these mutations also occur sporadically, giving us a chance to understand the role of these mutations in familial and at the same time sporadic cases, which represent the vast majority. The Catalogue of Somatic Mutations in Cancer (COSMIC) database have identified that 43% of cancer predisposition genes with germline mutations overlaps with somatic driver mutations^30^. Instead of reprogramming genetically unstable cancer cells with multiple mutations, tumor development could be studied in genetically stable cells carrying only what is thought to be the original driver mutation (Fig. 2b). Using this approach, iPS cells have been generated to model breast cancer (BRCA1 mutation) and Li Fraumeni syndrome (LFS; p53 mutation).

The tumor suppressors BRCA1/2 play an important role in DNA repair, and mutations in BRCA1/2 are found in familial as well as sporadic breast cancers. Even though mouse models have been informative, differences between mouse and human have to be taken into consideration. As an example, BRCA1 mutation in a single allele has been found to promote genomic instability in human cells but not in murine cells^31^. Therefore, iPS cell lines from a BRCA1-carrier with a 5382insC mutation, predicted to be high risk for breast and ovarian cancer, were successfully generated. Although the study did not investigate the cellular phenotype or tumorigenic potential of BRCA1 patient-derived iPS cells^32^, it encourages further use of this methodology for deeper understanding of BRCA-driven breast cancers. LFS is an autosomal dominant syndrome caused by mutations in the p53 tumor suppressor gene. LFS patients usually develop early onset of a variety of tumors including osteosarcoma (OS), breast cancer, sarcoma, and leukemia^33^. Non-cancerous fibroblasts from LFS patients with a germline p53 mutation (G245D), also commonly found in sporadic tumors, were reprogrammed into iPS cells. Since one of the major cancers affecting LFS families is OS, they went on to study the effect of this p53 mutation in osteoblasts (OB) differentiation. They found OB derived from LFS-iPS recapitulated OS characteristics, including defective differentiation and tumorigenic potential. Moreover, they found impaired expression of the imprinted H19 gene in LFS-derived OB and more interestingly, restoration of H19 expression improved OB differentiation. LFS mouse models have not been able to fully recapitulate the disease, however by using LFS patient-derived iPS cells they could recapitulate OS features and gene expression signatures and also identify significant dysregulated genes with potential clinical implications^34^.

In addition, other types of familial cancers could benefit from this type of modeling. For example, lynch syndrome or hereditary non-polyposis colorectal cancer (HNPCC), an autosomal dominant disorder is linked not solely to colon cancer but predisposes to ovarian, endometrium and other cancers that could also be studied using patient-derived lynch syndrome iPS cells. The DNA mismatch repair genes MLH1, MSH2, MSH6, and PMS2, are frequently mutated in HNPCC^35–37^. Importantly, the majority of these genes are also mutated in sporadic cancers; therefore, generating iPS cells from non-cancerous somatic cells carrying HNPCC-germline mutations would be the perfect alternative for studying not only colon cancer but also the contribution of these genes to other type of cancers. In addition, the differentiation potential of iPS cells into diverse cell lineages might undercover new biological functions of the familial mutated genes and their role in tissue-specific tumor development.

## Taking advantage of the developmental identity of iPS cells to create models for pediatric malignancies

One caveat of using iPS-derived cells to model cancer onset is the immature/young state of the derived cells. The reprogramming process resets the biological clock in the cells, acquiring an early embryonic identity rather than cells of an aging adult tissue^38^. This does not only pose a problem for cancer research but also for other age-related diseases such as neurodegeneration and cardiovascular disorders. For these reasons, several strategies have been developed for increasing the cellular age of iPS cells, including adding chemical compounds that accelerate differentiation and maturation^39^, transfections of genes that accelerate terminal differentiation^40^, or promote overall aging by increasing genomic instability (progeria accelerated cellular aging in vitro)^41^. However, considering that iPS cells and their derivatives in many aspects mimic early stages of human development, they are an attractive model for studying early onset diseases such as childhood cancers.

**Fig. 3:**
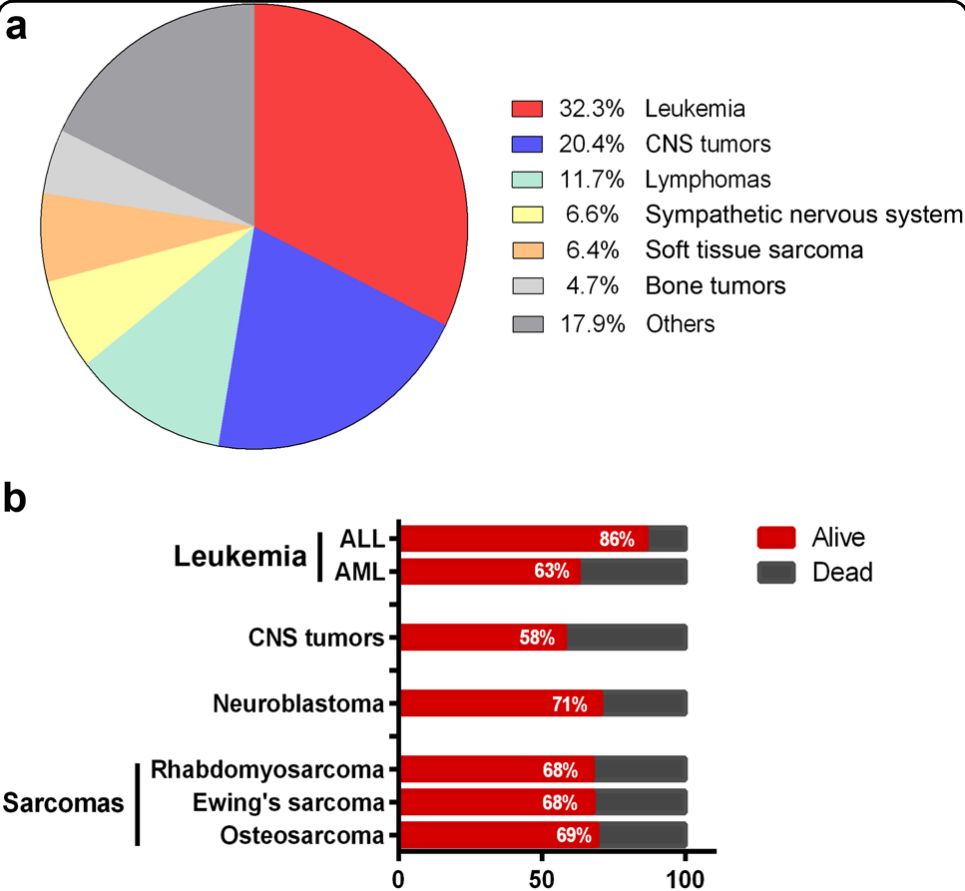
Childhood cancer incidence and survival **a** Global proportional distribution of childhood cancer incidence in age group 0–14 years, adapted from statistics presented in^42^. **b** 5-year survival of the most common forms of childhood cancer diagnosed in European patients during 2000–2007, adapted from the Eurocare-5 study^86^. ALL acute lymphoblastic leukemia, AML acute myeloid leukemia, CNS central nervous system

Although rare, childhood cancer is a major cause of death in children and adolescents’ worldwide. The most common cancer types in children are leukemia, CNS tumors, lymphomas, sarcomas, and peripheral nervous system tumors (Fig. 3a)^42^. Childhood cancers generally develop rapidly with fewer genetic aberrations compared to adult cancers, which is thought to be due to defects affecting stem or progenitor cells present during normal embryonic development. The 5-year all-cancer survival in children is around 80%, significantly better than for adults, however, certain cancer types such as pediatric CNS tumors have considerably lower survival rates (Fig. 3b). The vast majority of childhood cancers are treated with front lines therapies such as surgery, chemotherapy and radiotherapy, sometimes leaving the surviving children with serious side effects including hormone, growth, and cognitive disorders. In addition, in the last decade there has been very limited survival increases for a number of pediatric malignancies, suggesting that the present treatments have reach its limits^43^. It is therefore important to find new therapeutic options that provide better efficacy and fewer side effects. We suggest that reprogramming somatic non-cancerous cells from patients carrying cancer-predisposing germline mutations will generate new childhood cancer models and treatment options, and add valuable information of onset and development of childhood cancers. We will describe the recent advances and propose further direction in the most common types of childhood malignancies.

## Using iPS cells as a treatment option and model for childhood leukemia

Leukemias are the most common type of childhood cancers (Fig. 3a), and can develop from immature lymphoid progenitor cells (acute lymphoblastic leukemia, ALL), or from immature myeloid progenitor cells (acute myeloid leukemia (AML) and JMLL). ALL is the most common form of childhood leukemia, accounting for 80–90% of all leukemia cases, however, the mortality rate for AML is considerable worse compared to ALL, 63% vs. 86% (Fig. 3b), showing that more treatment options have to be developed for AML patients.

First-line treatment for leukemia often includes whole-body irradiation and transplantation of bone marrow-derived human hematopoietic stem cells (HSCs), peripheral blood, or umbilical cord stem cells. However, limitations arise due to low availability of human leukocyte antigen (HLA) match donors and low yield of HSCs for engraftment, decreasing the chance of successful treatments. The importance of correct donor-host HLA matching is crucial for avoiding graft-vs.-host disease and graft rejections^44^. Therefore, to be able to in vitro expand HSCs is an attractive treatment alternative for hematological diseases. In this case, human iPS cells could be used to supply infinitive in vitro production of distinct blood cell lineages and development of novel treatments. Numerous new protocols have been developed for generating hematopoietic cell lineages, including: human T and B cells^45–47^, megakaryocytes^45^, and erythroid cells^48,49^. However, generation of functionally mature cells and HSC with multi-lineage long-term engraftment potential (LT-HSC) is still a hurdle, although some advances have been made. HSCs isolated from iPSC-generated teratomas were capable of restoring the hematopoietic system of immunocompromised mice^50,51^. Also direct conversion from somatic cells to HSCs was attempted by using combination of specific TFs such as HoxB4^52,53^, panmyeloid (ETV2 and GATA2) and erythro-megakaryocytic (GATA2 and TAL1)^54^, although without the capability of long-term engraftment. However, the addition of strong TFs that directs differentiation towards a specific lineage also raises concerns for its use in a clinical setting. Recently, by direct conversion of iPS cells to hemogenic endothelium (HE)^55^ in combination with in vivo screening for specific HSCs factors, seven factors were found to be able to convert HE into HSCs that engrafted myeloid, B and T cells in mice and showed multi-lineage potential^56^, a promising step closer to new treatment options and more reliable models of hematological malignancies.

The most common germline mutations in familial leukemia are CEBPA, RUNX1, and GATA2^57^, so far there has been no attempt of reprogramming cells with these mutations. However, iPS cells have been generated from leukemia-causing somatic mutations. JMLL is initiated by deregulation of cytokine receptor signaling that causes enhanced myelopoiesis. Gandre-Babbe et al. generated iPS cells from malignant cells from two JMLL patients with somatic heterozygous missense mutations in PTPN11^26^, which encodes SHP-2, a non-receptor tyrosine phosphatase. These JMLL iPS cells could be differentiated into myeloid cells that behaved similarly to primary JMML cells, showing increased proliferative capacity, constitutive activation of granulocyte macrophage colony-stimulating factor (GM-CSF), and enhanced STAT5/ERK phosphorylation. Using iPS cell-derived myeloid cells for drug screening, MEK and JAK1/2 kinases inhibitors have been identified as new treatment options supporting clinical trials for MEK inhibition in JMLL patients.

Translocations at chromosome 11q23 involving the MLL gene is a frequent event in pediatric ALL and AML, and have been associated with an intermediate to poor outcome^58^. Recently it was reported that primary blasts from AML patients with MLL rearrangements could be reprogrammed into iPS cells. Patient-derived iPS cells reset epigenetic changes but retained the original genetic rearrangements. Interestingly, only AML-iPS cells that were differentiated into hematopoietic cells gave rise to leukemia in mice, whereas differentiation into other cellular lineages did not. In addition, they generated iPS cells from individual subclones from the AML patients that was used to design therapies targeting each subclone^59^. Moreover, Kotini et al. were able to show similarities by mimicking hematopoiesis to myelodysplastic syndrome and AML by introducing or correcting mutations using CRISPR–Cas9^60^.

## Modeling central and peripheral nervous system tumors

### Medulloblastoma

Brain tumors and other central nervous system (CNS) cancers are the second most common malignancies in children^61^. Medulloblastoma (MB) is one of the most frequent brain tumors, accounting for 20% of all brain tumors^62^. Despite advances in MB treatments have increased the survival rates in the past years^63^, post-treatment consequences can lead to severe and permanent neurocognitive defects^64^. Four sub-classifications can be distinguished within MB; wingless (WNT), sonic hedgehog (SHH) named for the signaling pathways primarily affected during MB pathogenesis, and group 3 and 4, where the pathogenesis of the last two groups is still currently unknown^65,66^.

### Medulloblastoma-associated hereditary syndromes

Genetic syndromes associated with MB are rare^67^. However, their molecular basis can be used to understand the onset and progression of this disease. The SHH and WNT-subtypes of MB constitute about 41% of total MB^68^. Germline mutations in SHH pathway components, *PTCH1*, *SMO* and *SUFU* predispose to different pathologies that include higher risk of MB^69^. Around 15% of sporadic MB cases presents any of this mutations^70^. Gorlin syndrome or nevoid basal cell carcinoma (NBCCS) is a hereditary disease caused by mutations in the *PTCH1* gene^71^. These patients develop different conditions, including basal cell carcinomas (BCC) and congenital skeletal abnormalities, but also other malignancies can be manifested such as MB^72^. Germline *SUFU* mutations also lead to infant desmoplastic MB^69^ and children develop similar symptoms as Gorlin patients^73^. Mutations in the *APC* gene are associated with familial adenomatous polyposis and Turcot syndrome. People with Turcot syndrome have an increased risk of developing MB. Modeling human MB using iPS cells from patients with MB-associated syndromes carrying any of these germline mutations could give new insights in how SHH and WNT signaling pathways contribute to MB and as well as initiation of other cancers with the same mutations. Importantly, the understanding of the role of these genes during normal human development is possible by using this type of approach^67,69,73,74^.

### Neuroblastoma

Neuroblastoma (NB) is an embryonic tumor believed to originate from neural crest cells during the development of the sympathetic nervous system. Tumors are often found in sympathetic nervous tissues such as the adrenal gland and paraspinal ganglia of young infants. Although NB incidence is relatively low, the 5-year mortality rate in children with high-risk NB is high and is mostly due to relapse cases^75^. Most typical genetic aberrations found in sporadic NB are copy-number alterations; MYCN amplifications, gain of chromosomes 11q, and 17q, and loss of 1p36, as well as different point mutations^76^. Although rare, germline mutations in anaplastic lymphoma kinase (ALK) and PHOX2B have been found in hereditary NB. PHOX2B, plays an important role during autonomic nervous system development, have been found involved in congenital central hypoventilation syndrome and associated with Hirschsprung disease^77^ and also found mutated in sporadic tumors^78^. Similarly, expression patterns of the tyrosine kinase receptor ALK suggest key roles in mammalian nervous system development^79^. ALK alterations have been described in multiple human cancers, most frequently translocations resulting in ALK fusion proteins in anaplastic large cell lymphoma, inflammatory myofibroblastic tumors and non-small cell lung cancers^80–82^. Identification of activating ALK point mutation in familial and sporadic NB opened up new possibilities for using targeted therapy such as Tyrosine kinase inhibitors. Here again, the use of iPS cells derived from patients carrying PHOX2B or ALK germline mutations could be used as models for understanding NB onset and development, other related syndromes and also their role in normal human development.

## Modeling sarcoma development

Pediatric sarcomas is a heterogeneous group of tumors that can be divided into soft tissue sarcomas, mainly rhabdomyosarcomas, and bone sarcomas, most common are Ewing’s sarcoma and osteosarcoma. Although present in young children, sarcomas are mainly occurring in adolescents and young adults. Primary treatment is surgery followed by chemotherapy, and although there has been a tremendous increase in survival the last decades there are still about one third of sarcoma patients that succumb to their disease (Fig. 3b). Several germline mutations have been shown to predispose to pediatric sarcoma development, including mutations in p53, PMS2, RET, RB1, and PALB2^83^.

In addition to the modeling of sarcoma development using iPS cells derived from Li Fraumeni patients or sarcoma cell lines, iPS cells derived from sarcoma patients have also been used to assess drug resistance mechanisms in Ewing’s sarcoma (EWS). The majority of EWS tumors carry the t(11;22)(q24;q12) chromosomal translocation generating the EWS–FLI1 fusion protein, a promiscuous TF that activates pro-oncogenic programs. Reprogramming of the Ewing’s sarcoma cell line, CHLA-10, into iPS cells (EWS-iPS) showed that although the expression of EWS–FL1 fusion transcript between CHLA-10 and EWS-iPS is similar, EWS-iPS developed resistance to compounds targeting EWS–FL1 driven pathways^84^. Interestingly, re-differentiation of EWS-iPS recovered drug sensitivity, suggesting that differentiation status is important for drug responses and the undifferentiated state of iPS cells could be used to study drug resistance mechanisms.

## Concluding remarks

The discovery of iPS cells opens up a wide spectrum of possible future applications including development of new treatments in regenerative medicine, generation of better and more accurate disease models, and improving drug discovery. Where other tools fail, using patient-specific iPS cells for modeling diseases have an incredible potential to improve our understanding of basic mechanisms operating during healthy and diseased human development and differentiation. With the development of precision editing techniques such as the CRISPR/Cas9 system there is now the possibility to tailor genomic modifications in iPS cell lines, giving new opportunities for disease modeling and for understanding the multi-step tumorigenic process. As proof of principle, with help of CRISPR/Cas9, mutations can be introduced or corrected and their effect on tumor development studied^60^. Cancer tissue specificity is an important issue to address. Germline mutations are present in every cell of the body, nevertheless, tumor development is usually restricted to a specific tissue or organ. Due to the differentiation potential of iPS cells, and the unlimited expandable source of material, it can be an important tool for understanding specific genetic mechanisms operating during this specification. In addition, transplanting disease-relevant cell types derived from patient iPS cells into an established microenviroment by using orthotopic xenograft models could generate new in vivo systems for modeling diseases.

Childhood cancers and familial cancers develop with shorter latency compared to adult sporadic tumors. It is thought that this is due to the transforming event coincides with mechanisms operating during normal human development. Taking advantage of the immature state of iPS cells would provide us with new potent models to study early events in cancer initiation and progression.
